# Host phylogeny and seasonality shapes avian haemosporidian prevalence in a Brazilian biodiverse and dry forest: the Caatinga

**DOI:** 10.1017/S0031182023000549

**Published:** 2023-12

**Authors:** Daniela de Angeli Dutra, Asmat U. Khan, Francisco C. Ferreira, Marina V. Beirão, Mauro Pichorim, Patrícia A. Moreira, Érika M. Braga

**Affiliations:** 1 Department of Zoology, University of Otago, 9016, Dunedin, Otago, New Zealand; 2 Departamento de Parasitologia, Instituto de Ciências Biológicas, Universidade Federal de Minas Gerais, Belo Horizonte, MG, Brazil; 3 Department of Zoology, Shaheed Benazir Bhutto University, Sheringal Dir Upper Khyber Pakhtunkhwa, Pakistan; 4 Department of Entomology, Texas A&M University, College Station, TX, USA; 5 Department of Veterinary Pathobiology, Schubot Centre for Avian Health, Texas A&M University, College Station, TX, USA; 6 Departamento de Biodiversidade, Evolução e Meio Ambiente, Instituto de Ciências Exatas e Biológicas, Universidade Federal de Ouro Preto, Ouro Preto, MG, Brazil; 7 Departamento de Botânica e Zoologia, Ciências de Biociências, Universidade Federal do Rio Grande do Norte, Natal, RN, Brazil

**Keywords:** Avian malaria, *Haemoproteus*, haemosporidian prevalence, host traits, *Plasmodium*, semiarid ecosystem

## Abstract

The relationships between host phylogenetics, functional traits and parasites in wildlife remain poorly understood in the Neotropics, especially in habitats with marked seasonal variation. Here, we examined the effect of seasonality and host functional traits on the prevalence of avian haemosporidians (*Plasmodium* and *Haemoproteus*) in the Brazilian Caatinga, a seasonally dry tropical forest. 933 birds were evaluated for haemosporidian infections. We found a high parasitism prevalence (51.2%), which was correlated with phylogenetic relatedness among avian species. Prevalence varied drastically among the 20 well-sampled species, ranging from 0 to 70%. Seasonality was the main factor associated with infections, but how this abiotic condition influenced parasite prevalence varied according to the host-parasite system. *Plasmodium* prevalence increased during the rainy season and, after excluding the large sample size of Columbiformes (*n* = 462/933), *Plasmodium* infection rate was maintained high in the wet season and showed a negative association with host body mass. No association was found between non-Columbiform bird prevalence and seasonality or body mass when evaluating both *Plasmodium* and *Haemoproteus* or only *Haemoproteus* infections. Parasite community was composed of 32 lineages including 7 new lineages. We evidenced that even dry domains can harbour a high prevalence and diversity of vector-borne parasites and pointed out seasonality as a ruling factor.

## Graphical abstract

**Figure:**
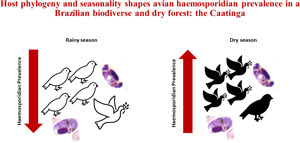

## Introduction

Seasonality is an important determinant of vector-borne diseases (Fecchio *et al*., 2019). Vectors, pathogens and hosts are dependent on abiotic conditions for reproduction and survival, and changes in these conditions may affect the transmission of many distinct diseases (Kelly-Hope *et al*., 2009; Gonzalez-Quevedo *et al*., 2014; Ferraguti *et al*., 2018). For instance, higher temperatures increase vector abundance and often accelerate parasite development in their vectors (Valkiūnas, 2005; Lapointe *et al*., 2010), thus likely increasing parasite prevalence in vertebrate hosts (Zamora-Vilchis *et al*., 2012). Similarly, heavy rain periods and severe droughts can increase or decrease the prevalence of diseases transmitted by vectors dependent on water collections for breeding sites (Hoshen and Morse, 2004; Landesman *et al*., 2007). Changes in the pattern of seasonality may become a challenge to future ecological studies due to global climate change (IPCC, 2021), thus it is important to understand the impact of seasonality on vector-borne pathogens distribution.

Avian haemosporidians are a diverse group of protozoan parasites, including the genera *Plasmodium* and *Haemoproteus* that use a variety of Diptera species as vectors. *Plasmodium* parasites are transmitted by mosquitoes (Culicidae) whereas *Haemoproteus* (*Parahaemoproteus*) and *Haemoproteus* (*Haemoproteus*) are transmitted by either biting midges (Ceratopogonidae) or louse flies (Hippoboscidae), respectively, hence, environmental conditions could affect their transmission differently (Ferreira *et al*., 2020). There are more than 200 species already described for these parasites, which can develop in a variety of bird and vector species (Marzal, 2012; Clark *et al*., 2014). Furthermore, avian haemosporidians are associated with mortality episodes in wild birds (Ricklefs, 2017) and can reduce longevity and reproductive fitness of chronically infected hosts (Marzal *et al*., 2005; Asghar *et al*., 2015).

Host biological and ecological traits also influence haemosporidian prevalence, diversity and distribution (Pulgarín-R *et al*., 2018; Fecchio *et al*., 2021; de Angeli Dutra *et al*., 2021*a*). Individual traits such as plumage colour and body mass are associated with differences in parasite prevalence (De La Torre *et al*., 2020; Filion *et al*., 2020). For example, a negative effect of haemosporidian parasites on body condition was detected among passerine species (Palinauskas *et al*., 2016; Schoenle *et al*., 2017). Species functional traits, such as habitat selection, plumage colouration, nest type, migratory behaviour and flocking, have all been implied as predictors in the variation of haemosporidian prevalence (Gonzalez-Quevedo *et al*., 2014; Ganser *et al*., 2020; de La Torre and Campião, 2021; de Angeli Dutra *et al*., 2021*b*; Aguiar de Souza Penha *et al*., 2022). For instance, *Haemoproteus* prevalence reaches higher rates among avian species inhabiting mid-high and canopy strata and haemosporidian infections are more common among strictly migratory species (de La Torre and Campião, 2021; de Angeli Dutra *et al*., 2021*b*). Moreover, species that are phylogenetically closely related tend to exhibit greater similarity in functional traits as compared to distantly related species, which may be correlated with parasite exposure and likelihood of infection (Barrow *et al*., 2019).

How seasonality affects avian haemosporidian parasites in tropical seasonally dry environments is still uncertain. Ferreira *et al*. (2017) found seasonal changes in parasite prevalence, while no variation was detected by Fecchio *et al*. (2015) These contrasting results indicate the need for further investigations of avian haemosporidian distributions across different periods in tropical areas with marked seasonality. The Caatinga is a seasonally dry tropical forest (SDTF) in Brazil. This domain is located exclusively in northeastern Brazil, covering an area of approximately 845 000 km^2^ which represents about 11% of the national territory (Bucher, 1982). The region's climate is classified as hot semi-arid (type ‘BSh’) according to Koöppen's classification (Alvares *et al*., 2013), characterized by a long dry season between July and January with irregular distribution of rainfall in the rest of the year. This domain, which had been considered inappropriately associated with low diversity regions in terms of endemism and species richness (Vanzolini *et al*., 1980; Leal *et al*., 2005), actually harbours high biodiversity. Caatinga is home to more than 200 bird species with 22 considered endemic (Tabarelli and Silva, 2003)

Due to the high biodiversity in the Caatinga and the lack of knowledge of the distribution and diversity of avian haemosporidians in this region and in seasonally dry environments, we aimed to investigate the effect of seasonality and host functional traits on avian haemosporidian prevalence and diversity in the Caatinga ecosystem.

## Methods

### Study area

**Figure 1.**
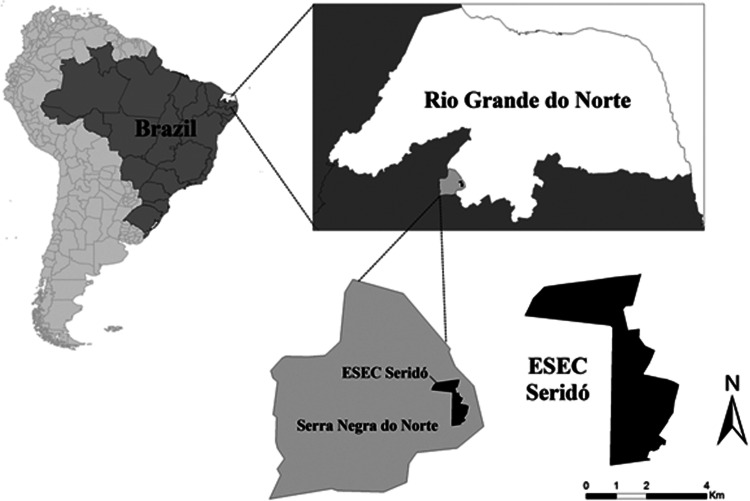
Map of Seridó Ecological Station (ESEC Seridó), Rio Grande do Norte, Brazil.

We conducted this study in Seridó Ecological Station – ESEC Seridó – (06° 34′36.2″ S and 37°15′20.7″ W), encompassing an area of 1163 ha and located in the municipality of Serra Negra do Norte, state of Rio Grande do Norte (Brazil) (Fig. 1). The region has a semiarid climate, with dry season reaching up to 10 dry months with irregular rainfall distribution. Mean annual precipitation varies between 500 and 800 mm year^−1^ and mean annual temperature varies between 28°C and 30°C, with lowest and highest temperatures ranging between 17°C and 40°C (Bucher, 1982). Local vegetation is composed of grass-covered soil and arboreal-shrub Caatinga with sparse small trees (<7 m) (Duque, 1953). The region has a high bird richness, with approximately 200 species, including some threatened and near-threatened species (Pichorim *et al*., 2016*a*, 2016*b*).

### Sample collection and DNA extraction

We captured wild birds in 4 field campaigns, each consisting of 7 days in 4 different sampling seasons (June 2013: ‘first rainy’, which had 45.8 mm of accumulated precipitation, January 2014: ‘second rainy’, with 95.4 mm of accumulated precipitation, July 2014: ‘first dry’ 7.0 mm of accumulated precipitation, and December 2014 ‘second dry’, no precipitation). Birds were captured using mist nets (Ecotone^®^; 18 m × 3 m, mesh 19 mm) set in a 12-ha quadrant (400 × 300 m). This large quadrant was divided into 48 cells measuring 50 × 50 m, with the capture site (i.e. where the mist net was placed) located at the centre of each cell. We sampled 24 cells per day between 5h00 and 10h00 in each field campaign, resulting in an effort of 181 440 m^2^ h (54 m^2^ of per net × 5 h per day × 24 nets per day × 7 days per campaign × 4 campaigns; Straube and Bianconi, 2002). Captured birds were identified, banded (with metal rings provided by CEMAVE/ICMBio (Centro Nacional de Pesquisa e Conservação de Aves Silvestres), weighed, and examined for the presence of ectoparasites (ticks, mites and lice) and brood patches. We collected blood samples from the brachial vein using insulin needles and stored the samples on filter paper. Captured birds were subsequently released near the capture sites. We extracted the genomic DNA from the blood samples using phenol-and Russellchloroform protocol followed by precipitation with isopropanol, as described by Sambrook and Russell (2001). We quantified the extracted DNA using NanoDrop™ Lite Spectrophotometer (Thermo Scientific^®^), according to the manufacturer's instructions.

### Molecular detection and characterization of haemosporidian parasites

We performed a screening PCR using primers designed by Fallon *et al*. (2003). To amplify both *Plasmodium* and *Haemoproteus* genera. All positive samples at the screening PCR were subjected to a Nested-PCR, described by Hellgren *et al*. (2004), which amplifies a 478 bp fragment of the mitochondrial cytochrome b gene (cyt-b) of *Plasmodium* and *Haemoproteus*. We did not perform the nested assay that amplifies *Leucocytozoon* parasites because of their low prevalence in Brazil (Fecchio *et al*., 2020). We used *Plasmodium gallinaceum* derived from experimentally infected chicks as a positive control. Sterile ultrapure water was used as a negative control. We performed all PCR and electrophoresis methods according to (Roos *et al*., 2015).

We purified the positive Nested-PCR products following (Green and Sambrook, 2012). The purified DNA was bi-directionally sequenced by the dideoxynucleotide method in ABI 3100^®^ capillary automated sequencer (Applied Biosystems, USA) using the Big Dye Terminator Mix kit (Applied Biosystems, USA) following reaction and reading conditions indicated by the manufacturer.

We edited obtained sequences using Chromas Pro (Technelysium Pty Ltd, Helensvale, Australia) checking for the presence of mixed infections (presence of double peaks in the eletrochromatograms). We compared our assembled sequences to those deposited in public databases, such as GenBank (http://www4.ncbi.nlm.nih.gov) and MalAvi (Bensch *et al*., 2009). Sequences with a minimum of one base difference were considered unique cytochrome b lineages, and those with no database record were considered novel lineages. We deposited novel lineages in GenBank (acc. num.MK981615–MK981622). New records of previously described sequences were also deposited in GenBank (acc. num. MK981623–MK981646).

### Host functional traits data

We obtained avian functional trait data for each host from AVONET (Tobias *et al*., 2022). We included the variables and categories as follows: (1) migratory behaviour: resident, partially migratory and strictly migratory; (2) primary lifestyle: insessorial, terrestrial and generalist; (3) body mass; (4) host distribution range (i.e. geographical distribution of a bird species).

### Statistical analysis

#### Phylogenetic signal

All analyses were conducted in R version 4.0 (R Core Team, 2017). Firstly, we filtered all bird species that were sampled 4 or fewer times, this filtered dataset (*N* = 880) was used in all following analyses. To evaluate if the phylogenetic relationship among bird species is correlated with parasite prevalence in our dataset, we downloaded a full avian phylogeny file from the AllBirdsHackett1.tre website (https://birdtree.org/) which contain 10 thousand trees (Hackett *et al*., 2008; Jetz *et al*., 2012). Later, we applied the ‘treeman’ package (Bennett *et al*., 2017) to create a treeman file containing all trees from the original file. Then, we randomly selected a phylogenetic tree to avoid selection bias. We excluded all bird species from the tree which were not present in our dataset. Then, we calculated *K* (i.e. a measure that allows comparisons of the amount of phylogenetic signal across a specific trait (Blomberg *et al*., 2003)) to evaluate the phylogenetic signal for haemosporidian prevalence among bird species in our dataset. Values of *K* can range between 0 and 1, equalling 1 when the trait has evolved consistently with a Brownian motion and trait values are similar among related species, or 0 when trait values are phylogenetically unrelated among species. To estimate *K*, we applied the ‘phylosig’ function from the ‘phytools’ package (Revell, 2012).

#### Bayesian analyses

We constructed 4 Bayesian models using the ‘brms’ package (Bürkner, 2017) to evaluate whether bird functional traits and seasonality influence haemosporidian prevalence. In all models, we created a matrix with phylogenetic distances among all avian species to account for influence of host phylogenetic relationships on haemosporidian prevalence, which was included as a random variable. Since we observed strong phylogenetic signals in our dataset (see Results), adding phylogenetic relatedness among species in our models was important to take into consideration when evaluating the effect of the other variables included in the models. The explanatory variable effects included in each model are represented in Table 1. The 4 models were weighted using the function ‘loo_model_weights’ and the one with the highest weight value was selected. In all models, we used the infection status of individual birds as our dependent variable (binary response: 0 for uninfected, 1 for infected). We ran all models using the Bernoulli distribution family and 4 chains with 4000 total iterations per chain (2000 for warmup, 2000 for sampling). Priors were chosen using ‘get_prior’ function and the models' results were plotted using the ‘conditional_effects’ function to visualize the predictions of the population-level effects. The selected model was repeated 6 times using haemosporidians of both genera, *Plasmodium* only and *Haemoproteus* (both subgenera) only parasites (whenever the parasite ID was achieved through sequencing) and using the entire dataset (*N* = 880) or the dataset excluding Columbiformes (*N* = 483), which represented most of the birds sampled. It is imperative to rerun analyses excluding Columbiformes due to the minor influence of environmental conditions on *H. (Haemoproteus)* vectors, as those mostly reside on their hosts' skin and Columbiformes are the main hosts of *H. (Haemoproteus).*

**Table 1.** Model averaging weights for the 4 models tested using pseudo-BMA (where higher values indicate better model fit) with Bayesian bootstrap method

*Models*	*Pseudo-BMA*
*Infection status* ~ *Primary.Lifestyle + Incubation + Ectoparasites + Season + Mass + Migration + Range.Size*	0.0083
*Infection status* ~ *Primary.Lifestyle + Incubation + Ectoparasites + Season + Mass + Migration*	0.103
*Infection status* ~ *Incubation + Ectoparasites + Season + Mass + Migration*	0.246
*Infection status* ~ *Incubation + Ectoparasites + Season + Mass*	0.563

## Results

### Plasmodium and haemoproteus diversity

We detected 481 positive samples (prevalence equal to 51.2%) in the screening PCR. Haemosporidian prevalence varied drastically among the 20 best sampled host species, ranging from 0 to 70% (Supplementary Table 2). All of those were subjected to cyt-*b* PCR and gene sequencing. However, we were able to obtain high-quality sequences for 191 individuals, which revealed 68 *Plasmodium* infections in 22 bird species (38.41%), 90 subgenus *Haemoproteus* (*Haemoproteus*) infections in 4 bird species (50.84%), and 19 subgenus *Haemoproteus* (*Parahaemoproteus*) infections in 10 bird species (10.83%). We were able to separate out haplotypes in 14 mixed infections in 10 bird species (3.92%), revealing *Plasmodium/Plasmodium* (*n* = 5), *H.* (*Haemoproteus*)*/H.* (*Haemoproteus*) (*n* = 7) and *H.* (*Parahaemoproteus*)*/H.* (*Parahaemoproteus*) (*n* = 2) infections. The parasite community was composed of 32 distinct lineages (*Plasmodium* = 17; *H.* (*Haemoproteus*) = 05 and *H.* (*Parahaemoproteus*) = 10); 7 haemosporidian lineages were obtained for the first time.

We observed a difference in host range among distinct parasite taxa. *Haemoproteus* (*Haemoproteus*) mainly infected Columbiformes (88/90; 04 species), with 2 lineages detected in passerines; SocH3 infected *Pachyramphus polychopterus* (Tityridae) and SocH2 *Myiarchus tyrannulus* (Tyrannidae). This parasite subgenus was represented by 5 genetic lineages: SocH3 (*n* = 69), COPIC01 (*n* = 16), SocH2 (*n* = 3) and by 2 new lineages ZENAUR01 (*n* = 1) and ZENAUR02 (*n* = 2). We obtained 68 sequences of genus *Plasmodium* representing 17 lineages, including three novel one (POLPLU01, PHAMUR01 and NYSMAC05), that mainly infected Passeriformes. The most common lineages were PADOM11 (detected 15 times in 6 bird species), PHPAT01 (13 times in 9 species) and PADOM09 (10 times in 8 species). *Haemoproteus* (*Parahaemoproteus*) parasites were found 19 times, and the most common lineage, PAPOL03, was observed 8 times in 4 bird species. All haemosporidian-host links are available in Supplementary Table 3.

### Factors influencing haemosporidian prevalence

**Figure 2.**
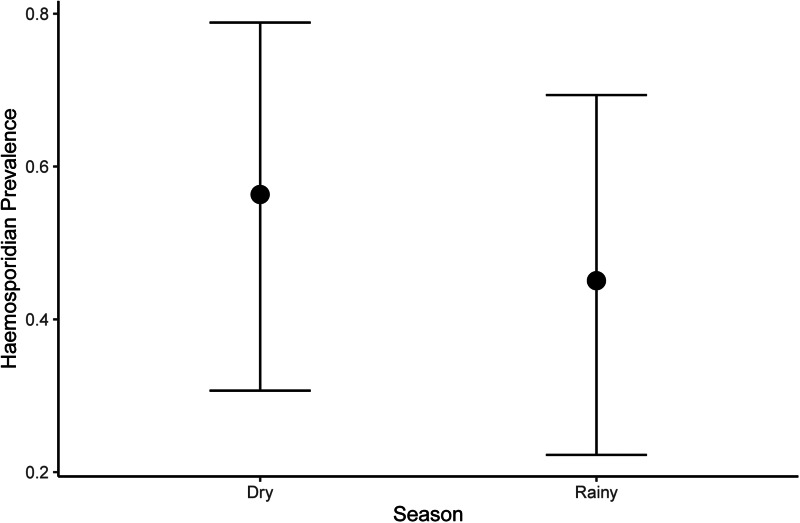
Mean (±credible intervals) haemosporidian prevalence according to season birds were collected.

**Figure 3.**
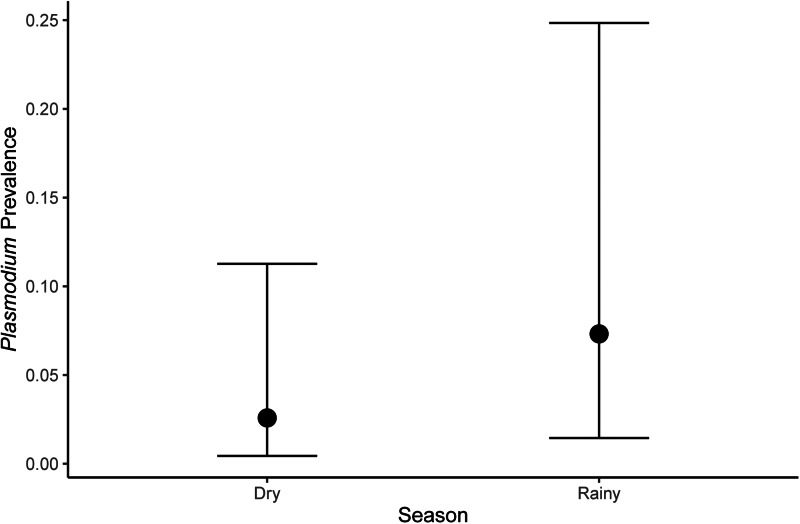
Mean (±credible intervals) *Plasmodium* prevalence according to season birds was collected.

**Figure 4.**
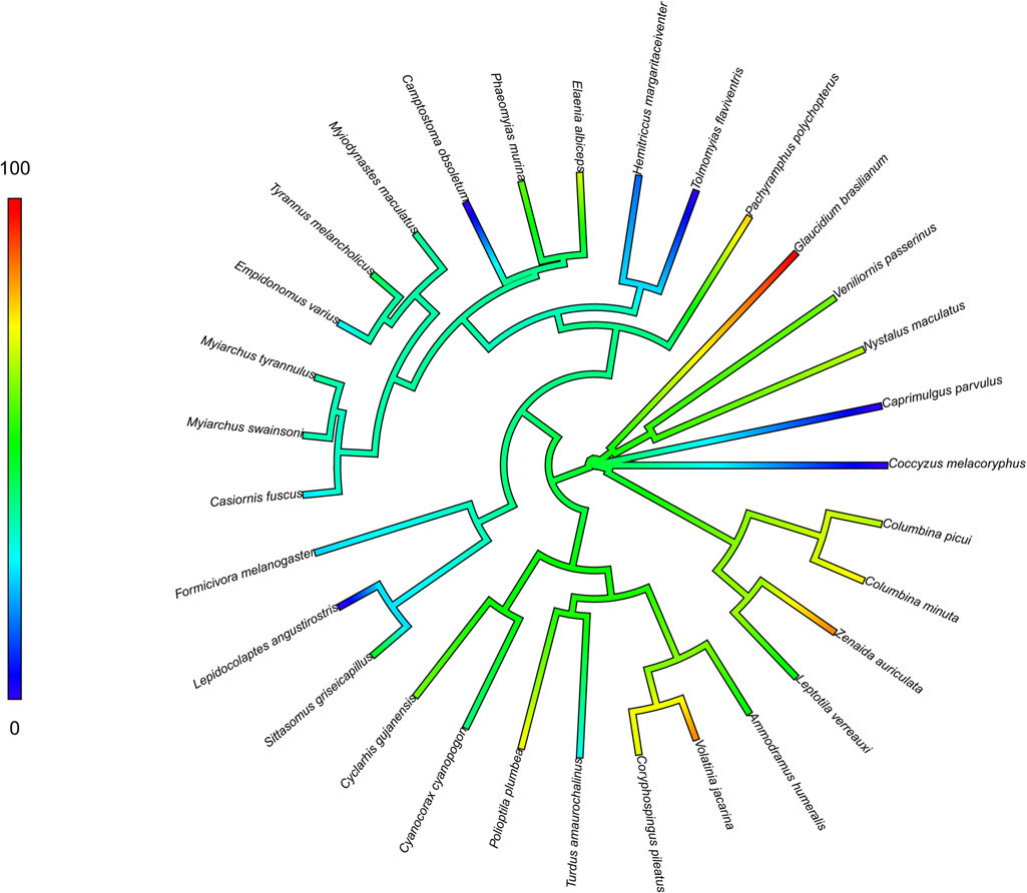
Mean prevalence according to the phylogenetic relationships across all bird species with more than 10 individuals sampled (*n* = 32).

We observed that the haemosporidian prevalence was influenced by seasonality and body mass (Tables 2–4). However, the way that the seasonality influenced parasite prevalence varied according to the haemosporidian genus. When examining the entire dataset, we observed higher haemosporidian prevalence during the dry season (Fig. 2), whereas for *Plasmodium* we found higher prevalence in the rainy season (Fig. 3) and found no difference among seasons when looking at *Haemoproteus* parasites separately (Table 3). Likewise, when evaluating only non-Columbiform birds, we still observed higher *Plasmodium* prevalence in the rainy season. Nonetheless, seasonality did not influence overall haemosporidians prevalence and when *Haemoproteus* was analysed separately (Table 4). We also observed that Columbiform species were more common in the dry season, when they represented 66% of the sampled birds, compared to only 31% of the sampled birds during the rainy season. Further, haemosporidian prevalence varied between Columbiform and non-Columbiform species, being 61% for Columbiformes and 42% for other birds. Among non-Columbiformes birds, body mass was negatively associated with infection when evaluating *Plasmodium* prevalence among different species (Table 4B). We did not observe correlation between most bird functional traits and haemosporidian prevalence in our dataset, but overall prevalence varied according to phylogenetic relatedness among avian species (*K* = 0.67, Fig. 4).

**Table 2.** Summary information of the sampling seasons, including infection data

Sampling/Season	Positive	Negative	Total	Prevalence
First rainy	109	129	238	45.79%
Second rainy	99	114	213	46.47%
First dry	197	127	324	60.80%
Second dry	76	82	158	45.79
Total	481	528	933	51.5%

**Table 3.** Estimate, standard error and credible intervals for A- haemosporidians, B- *Plasmodium* and C – *Haemoproteus* prevalence and host functional traits and seasonality

*A – All infections*	*Estimate*	*Error*	*Credible intervals*	
*Intercept*	0.26	0.53	−0.81	1.32
*Incubation stage 1*	0.26	0.47	−0.67	1.18
*Incubation stage 2*	1.23	0.45	−0.19	2.78
*Ectoparasites*	−0.12	0.20	−0.50	0.28
*Season (Rainy)*	−0.46	0.22	−0.89	−0.04
*Mass*	−0.10	0.16	−0.41	0.21
*B – Plasmodium*	Estimate	Error	Credible intervals	
*Intercept*	−3.66	1.08	−5.79	−1.45
*Incubation stage 1*	−0.39	0.80	−2.07	1.05
*Incubation stage 2*	0.79	0.86	−1.01	2.41
*Ectoparasites*	−0.14	0.36	−0.84	0.56
*Season (Rainy)*	1.09	0.46	0.21	2.04
*Mass*	0.18	0.26	−0.72	0.30
*C – Haemoproteus*	Estimate	Error	Credible intervals	
*Intercept*	−3.44	1.08	−5.79	−1.45
*Incubation stage 1*	0.89	0.84	−0.81	2.50
*Incubation stage 2*	1.48	1.13	−0.81	3.59
*Ectoparasites*	−0.15	0.31	−0.77	0.46
*Season (Rainy)*	0.23	0.33	−0.41	0.88
*Mass*	0.09	0.32	−0.58	0.71

**Table 4.** Estimate, standard error and credible intervals for A- haemosporidians, B- *Plasmodium* and C – *Haemoproteu*s prevalence and host functional traits and seasonality excluding Columbiformes hosts.

*A – All infections*	*Estimate*	*Error*	*Credible intervals*	
*Intercept*	0.00	0.74	−1.47	1.47
*Incubation stage 1*	0.23	0.49	−0.72	1.19
*Incubation stage 2*	1.23	0.78	−0.28	2.84
*Ectoparasites*	−0.03	0.25	−0.51	0.47
*Season (Rainy)*	−0.37	0.32	−1.00	0.24
*Mass*	−0.18	0.23	−0.65	0.28
*B – Plasmodium*	Estimate	Error	Credible intervals	
*Intercept*	−3.61	0.82	−5.34	−2.14
*Incubation stage 1*	−0.38	0.77	−2.00	1.04
*Incubation stage 2*	0.68	0.85	−1.07	2.27
*Ectoparasites*	−0.09	0.38	−0.82	0.65
*Season (Rainy)*	0.95	0.48	0.04	1.91
*Mass*	−1.38	0.54	−2.52	−0.41
*C – Haemoproteus*	Estimate	Error	Credible intervals	
*Intercept*	−4.89	1.49	−8.07	−2.15
*Incubation stage 1*	0.43	1.00	−1.67	2.27
*Incubation stage 2*	1.21	1.15	−1.14	3.36
*Ectoparasites*	0.42	0.55	−0.64	1.55
*Season (Rainy)*	0.93	0.92	−0.69	2.92
*Mass*	−0.14	0.57	−1.40	0.83

## Discussion

Investigating patterns and functional traits associated with infection is primordial to understand parasite infection dynamics and to determine main target species for conservation programs. Here, we reported that haemosporidian prevalence follows a seasonal pattern and varies distinctively among parasite taxa. Interestingly, we observed that body mass was negatively associated with *Plasmodium* prevalence among non-Columbiform birds, which contradicts a global analysis showing that infection probability for *Plasmodium* is higher in hosts with larger body (Gutiérrez-López *et al*., 2019; Filion *et al*., 2020). Moreover, we also evidenced a very high-level of phylogenetic association between haemosporidian prevalence and birds from the Brazilian Caatinga.

Phylogenetic relationships among hosts often reflect their association with parasites (Clark *et al*., 2018; Pacheco *et al*., 2018; Park *et al*., 2020; de Angeli Dutra *et al*., 2022). For this reason, parasite prevalence might vary following phylogenetic relationships among hosts (i.e. closely related hosts present more similar infection rates than distantly related ones), was also observed in this study. Parasites often perform well (i.e. are more successful in completing their life cycle to then be detected in the blood stream) among closely related hosts (Pinheiro *et al*., 2016), hence, similarity in prevalence among related species should reflect a tendency of those species to support the development of similar parasites lineages. Consequently, our results reinforce the fact that closely related hosts harbour similar prevalence patterns within a community. Most importantly, our results also evidence that the high abundance of Columbiformes species observed in the Caatinga could explain the uncommonly high prevalence of *Haemoproteus* parasites observed in this study in comparison to previous studies showing that *Plasmodium* is the most prevalent haemosporidian genus in Brazil (Lacorte *et al*., 2013; Ferreira *et al*., 2017; Rodrigues *et al*., 2021) and in the Neotropics (Fecchio *et al*., 2021).

Climatic conditions often affect the transmission of vector-borne pathogens due to direct effects on their vectors' abundance and diversity (Consoli and Oliveira, 1994). For this reason, regions presenting high seasonality might be subject to seasonal changes in parasite prevalence and incidence (Lalubin *et al*., 2013; Ferreira *et al*., 2017). It occurs due to seasonal changes in vector composition and abundance related to precipitation and temperature that are positively associated with vector abundance (Lalubin *et al*., 2013; Ferreira *et al*., 2016). Indeed, the rainy season harbours higher abundance of haemosporidian vectors in the Caatinga (Vasconcellos *et al*., 2010) and greater prevalence of *Plasmodium*. Nonetheless, when analysing haemosporidians in general we observed higher prevalence during the dry season in Caatinga, which could be due to the higher proportion of Columbiform birds in the dry season and *H.* (*Haemoproteus*) infections in those hosts.

We found a high haemosporidian prevalence in Caatinga (51%) compared to other Brazilian domains, such as 27–42% in the Brazilian savannah (Lacorte *et al*., 2013; Ferreira *et al*., 2017), 25–33% in the Atlantic rainforest (Lacorte *et al*., 2013; Rodrigues *et al*., 2021), and 20% in the Amazon rainforest (Fecchio *et al*., 2017). *H. (Haemoproteus)* represented most of the infections (50.9%), followed by *Plasmodium* (38.4%) and *H. (Parahaemoproteus)* (10.8%). In most studies conducted in Brazil, however, *Plasmodium* parasites were the most common. This different scenario in parasite prevalence may be explained by the abundance of *Columbina picui*, which harbours a high prevalence of *H.* (*Haemoproteus*) parasites. High levels of infection among Columbiformes birds by *H. (Haemoproteus)* might be associated with its vector biology since those flies (Hippoboscidae) spend nearly their entire adult life on their hosts (Valkiūnas, 2005). Changes in bird composition in Caatinga have been associated with low precipitation levels, which can trigger migratory movements among several species, increasing the relative proportion of resident species (Pereira, 2013).

Moreover, a high parasite richness in the Caatinga was observed in this study (*Plasmodium* = 17; *H.* (*Haemoproteus*) = 5 and *H.* (*Parahaemoproteus*) = 10) and 7 new parasite lineages were described. Both *Plasmodium* and *H.* (*Parahaemoproteus*) lineages infected a high number of bird species (22 and 10 species, respectively) while *H.* (*Haemoproteus*) only infected 4 bird species. This high parasite diversity and endemism (21% of all parasite lineages) in the Caatinga reveals the importance of studies in areas with a high degree of host endemism. Moreover, we detected *H.* (*Haemoproteus*) infecting Passeriform birds (SocH2 from *M. tyrannulus* and SocH3 from *P. polychopterus*). This parasite group is known to only infect a few seabird species and birds from the order Columbiformes (Levin *et al*., 2012). Parasite lineages from this subgenus have also been found infecting passerine birds in 2 other studies conducted in Brazil (Lacorte *et al*., 2013; Ferreira *et al*., 2017). These findings highlight that non-Columbiformes are exposed to parasites belonging to the subgenus *H.* (*Haemoproteus*) this SDTF. However, this likely represents abortive infections, i.e. infections in which the parasite cannot complete its lifecycle (Valkiūnas *et al*., 2009). Overall, we found high diversity of haemosporidian parasites in the Caatinga, which infections are mostly represented by *H.* (*Haemoproeus*) parasites, unlike most other regions from Brazil and South America.

To conclude, haemosporidian prevalence in the Caatinga seems to be higher than in other Brazilian biomes. Prevalence varied between dry and rainy seasons depicting higher prevalence during the dry season. *Plasmodium* relative frequency was higher in the rainy season while *H. (Haemoproteus)* was more frequent during the dry season. Our models showed that seasonality was the main factor associated with haemosporidian infections, however, it affected distinct parasite genera differently. This is one of the first studies conducted in a SDTF in South America, the Caatinga, which harbours a high diversity and a considerable prevalence of haemosporidians parasites. However, it is important to note that this study comprises only 1 locality in the Caatinga and that several haemosporidian infections lacked parasite identification. For this reason, further studies with diverse host species comprising multiple locations may reveal the uncovered diversity and possible endemicity of haemosporidian lineages in Caatinga.

## Supporting information

de Angeli Dutra et al. supplementary material 1 de Angeli Dutra et al. supplementary material

de Angeli Dutra et al. supplementary material 2 de Angeli Dutra et al. supplementary material

de Angeli Dutra et al. supplementary material 3 de Angeli Dutra et al. supplementary material

## Data Availability

Data used to perform this research is available as supplementary information or can be shared by Prof. Érika Martins Braga upon reasonable request.

## Supplementary material

The supplementary material for this article can be found at https://doi.org/10.1017/S0031182023000549
